# Supplementary material for: Drug supply management at first-level public health facilities: Case of Pyay District, Myanmar
Source: PLOS Glob Public Health. 2024 Sep 10;4(9):e0003692. doi: 10.1371/journal.pgph.0003692 (PMC11386443; doi:10.1371/journal.pgph.0003692)
Supplement: Attachment — Submitted filename: Response to Reviewers.doc [file pgph.0003692.s001.doc]

**Drug Supply Management at First-level Public Health Facilities: Case of Pyay District, Myanmar**

**Response to Reviewers**

| Number | Original comments of the reviewer | Reply by the author(s) | Changes done on the page number and line number |
| --- | --- | --- | --- |
| 17 July 2024 | | | |
| 1 | Tables should not be uploaded as individual files. Please remove these files and include the Tables in your manuscript file as editable, cell-based objects. | The authors have already uploaded all includable tables in the Manuscript and made all tables editable and cell-based objects. | Table 1. (Line number -263)  Table 2. (Line number -337)  Table 3. (Line number -353)  Table 4. (Line number -382)  Table 5. (Line number -421)  Table 6. (Line number -449)  Table 7. (Line number -486)  Table 8. (Line number -488)  Table 9. (Line number -518)  Table 10. (Line number -538)  Table 11. (Line number -558)  Table 12. (Line number -585)  Table 13. (Line number -602)  Table 14. (Line number -637) |
| 2 | Any typographical or grammatical errors should be corrected at the revision | The authors have revised the typographical or grammatical errors of the whole manuscript. | Whole manuscript. |
| 3 | I'd like for the authors to review all the abbreviations. Each abbreviation should be defined only once, at its first appearance in the manuscript (from introduction to conclusion). Subsequently, only the short forms should be used. | The authors revised all abbreviations as recommended by the reviewers. | AIDS – (Line number -64)  RHC and Sub-RHC (Line numbers 85-86)  NSCMS (Line number -91)  CMSD (Line number -118)  TB, HIV, NCD (Line numbers -148-149)  RHC and Sub-RHC (Line numbers 153-154)  OPD and NCD (Line numbers -158)  WHO, CHD and BASICS (Line numbers -178-179)  SOPs (Line number -221)  LMIS (Line number -231)  SPSS (Line number -308)  MCH, RHC and Sub-RHC (Line numbers 358-359)  PHS-1, PHS-2, LHV and HA (Table -2) |
| 4 | The background is very vast and can be shortened. | The authors removed some information from the introduction as recommended by the reviewers | Line numbers 132- 134. |
| 5 | The author should mention how these health facilities were selected and clearly describe the sampling method. | The authors have already mentioned " The study targeted all 43 RHCs, 6 Maternal and Child Health Centers (MCHs), and 134 Sub-RHCs, totalling 183 public health facilities within Pyay District, Bago Region. Face-to-face interviews were conducted with the health personnel managing the drug stores (a total of 183) from each RHC and Sub-RHC."  The sampling method is not eligible because the authors selected all health facilities and drug store management staff from the study area. | Line numbers -153-156. |
| 6 | Have you validated your data collection tools? | To validate the data collection tool, the authors conducted a pilot study before data collection. The authors have mentioned about the pilot study like " A pilot study was conducted in selected RHC and Sub-RHC within Nattalin Township, Bago Region, to assess the feasibility of data collection instruments, evaluate the data collectors' comprehension of methods and procedures, and estimate the time required for data collection." | Line numbers 291-294. |
| 7 | Various functional areas, such as the capability maturity level of capacity-building in drug supply management, the capability maturity level of drug forecasting and planning etc. can be expressed as outcome measures and written in the form of a table. | As recommended by the reviewers, the authors have explained the capability maturity level of the drug supply chain in the form of a table. | Line numbers -201. |
| 8 | The time period of data collection needs to be mentioned under the heading of data collection. | The authors mentioned the date of data collection " The data collection was started on 31^st^ March 2023 and ended on 9^th^ May 2023." | Line number -296. |
| 9 | This paragraph needs to be shortened. There isn't a need to explain everything. Only the ethical approval number and details of the source of ethical approval are enough. | **The authors** have revised the ethical consideration " The research adhered to strict ethical standards, receiving approval from the Institutional Review Board (Nay Pyi Taw), Ministry of Health, Myanmar. Participation was voluntary, with informed consent obtained through detailed forms translated into Myanmar and explained verbally when necessary. Anonymity and confidentiality were maintained, with data securely stored for five years and restricted access to authorized personnel. Results were presented accurately, and precautions were taken to avoid harm during data collection. The study, approved under IRB number 2023-06, emphasized ethical practices throughout its execution." | Line numbers -317-323. |
| 10 | Result texts need to be shortened. Information written as texts, is already there in tables therefore duplication should be avoided. | The authors summarized all texts in the findings as recommended by the reviewers. | Line numbers -367-695. |
| 22 July 2024 | | | |
| 11 | Please send a completed 'Competing Interests' statement, including any COIs declared by your co-authors. If you have no competing interests to declare, please state "The authors have declared that no competing interests exist". | The authors have already mentioned such a claim " The authors have declared that no competing interests exist" in both of manuscript and online submission. | Line number -1057. |
| 12 | Please amend your detailed Financial Disclosure statement. This is published with the article. It must therefore be completed in full sentences and contain the exact wording you wish to be published.  a. State the initials, alongside each funding source, of each author to receive each grant. For example: ""This work was supported by the National Institutes of Health (####### to AM; ###### to CJ) and the National Science Foundation (###### to AM).""  b. State what role the funders took in the study. If the funders had no role in your study, please state: “The funders had no role in study design, data collection and analysis, decision to publish, or preparation of the manuscript.” | The authors have already mentioned such claims " The required funds for conducting this research were granted by Ministry of Health and Sports (MOHS) Research Grant, Myanmar, (2022-2023). The grant number is 089/2022/MOHS. Date of receiving grant is 2 December 2022. The research grant is only for the study. The funder had no role in the study design, data collection and analysis, decision to publish, or preparation of manuscript." In the online submission and " The required funds for conducting this research were granted by MOHS Research Grant (2022-2023)**.** The funder had no role in the study design, data collection and analysis, decision to publish, or preparation of manuscript." In the manuscript. | Line number -1054-1055. |
| 13 | In the online submission form you indicate that your data is not available for proprietary reasons and have provided a contact point for accessing this data. Please note that your current contact point is a co-author on this manuscript. According to our Data Policy, the contact point must not be an author on the manuscript and must be an institutional contact, ideally not an individual. Please revise your data statement to a non-author institutional point of contact, such as a data access or ethics committee, and send this to us via return email. Please also include contact information for the third party organization, and please include the full citation of where the data can be found. | The authors changed the information about the data availability to " All collected data and synthesized information were displayed in the forms of words and tables in the finding portion and more information can be enquired from the Institutional Review Board, Naypyitaw, Myanmar, irb1@mohs.gov.mm." | Line number -1041-1043. |
| 14 | Please upload a clean revised manuscript with no tracked changes. | The authors re-submitted a clean revised manuscript with no tracked changes. |  |
